# Solid Self-Nanoemulsifying Drug Delivery Systems of Furosemide: In Vivo Proof of Concept for Enhanced Predictable Therapeutic Response

**DOI:** 10.3390/ph17040500

**Published:** 2024-04-14

**Authors:** Sania Gul, Sathvik Belagodu Sridhar, Aamir Jalil, Muhammad Akhlaq, Muhammad Sohail Arshad, Hafiz Shoaib Sarwar, Faisal Usman, Javedh Shareef, Sabin Thomas

**Affiliations:** 1 Department of Pharmaceutics, Faculty of Pharmacy, Bahauddin Zakariya University, Multan 60800, Pakistansohailarshad@bzu.edu.pk (M.S.A.); faisal.usman@bzu.edu.pk (F.U.); 2 RAK College of Pharmacy, RAK Medical & Health Sciences University, Ras al Khaimah 11172, United Arab Emirates; sathvik@rakmhsu.ac.ae (S.B.S.); javedh@rakmhsu.ac.ae (J.S.); 3 Department of Pharmacy, Hazara University, Mansehra 21300, Pakistan; makhlaq@hu.edu.pk; 4 Department of Pharmaceutical Sciences, University of Central Punjab, Lahore 54590, Pakistan; shoaib.sarwar@ucp.edu.pk; 5 College of Health Sciences, University of Nizwa, Birkat Al Mouz, Nizwa 616, Oman; sabin@unizwa.edu.om

**Keywords:** intestinal permeation, lipase activity, diuretic activity, mucus diffusion, solid SNEDDS

## Abstract

Liquid self-nano emulsifying drug delivery systems (SNEDDS) of furosemide (FSM) have been explored as a potential solution for enhancing solubility and permeability but are associated with rapid emulsification, spontaneous drug release, and poor in vivo correlation. To overcome the shortcoming, this study aimed to develop liquid and solid self-emulsifying drug delivery systems for FSM, compare formulation dynamics, continue in vivo therapeutic efficacy, and investigate the advantages of solidification. For this purpose, liquid SNEDDS (L-SEDDS-FSM) were formed using oleic acid as an oil, chremophore EL, Tween 80, Tween 20 as a surfactant, and PEG 400 as a co-surfactant containing 53 mg/mL FSM. At the same time, solid SNEDDS (S-SEDDS-FSM) was developed by adsorbing liquid SNEDDS onto microcrystalline cellulose in a 1:1 ratio. Both formulations were evaluated for size, zeta potential, lipase degradation, and drug release. Moreover, in vivo diuretic studies regarding urine volume were carried out in mice to investigate the therapeutic responses of liquid and solid SNEDDS formulations. After dilution, L-SEDDS-FSM showed a mean droplet size of 115 ± 4.5 nm, while S-SEDDS-FSM depicted 116 ± 2.6 nm and zeta potentials of −5.4 ± 0.55 and −6.22 ± 1.2, respectively. S-SEDDS-FSM showed 1.8-fold reduced degradation by lipase enzymes in comparison to L-SEDDS-FSM. S-SEDDS-FSM demonstrated a sustained drug release pattern, releasing 63% of the drug over 180 min, in contrast to L-SEDDS-FSM, exhibiting 90% spontaneous drug release within 30 min. L-SEDDS-FSM exhibited a rapid upsurge in urine output (1550 ± 56 μL) compared to S-SEDDS-FSM, showing gradual urine output (969 ± 29 μL) till the 4th h of the study, providing sustained urine output yet a predictable therapeutic response. The solidification of SNEDDS effectively addresses challenges associated with spontaneous drug release and precipitation observed in liquid SNEDDS, highlighting the potential benefits of solid SNEDDS in improving the therapeutic response of furosemide.

## 1. Introduction

Furosemide (FSM) is a BCS class IV potent loop diuretic, providing challenges for oral administration due to its low solubility and low permeability, resulting in unreliable bioavailability and an inconsistent therapeutic response [1]. Erratic absorption and variable blood serum concentration abruptly disturbed electrolyte balance and caused hypovolemic shock due to excessive irregular diuresis, potentially causing life-threatening situations [2]. Only a few approaches to drug delivery are available to overcome poor aqueous solubility and erratic absorption.

Among these strategies, lipid-based delivery systems are considered crucial formulation approaches to address unpredictable bioavailability problems. Such lipid formulations enhance drug solubility by producing a concentration gradient that facilitates drug absorption from potential intestinal absorptive sites [3]. SNEDDS, being liquid isotropic mixtures of oils, surfactants, and co-surfactants, are the most investigative and practical approach among lipid-based formulations, creating an effect similar to that of lipidic foods that are rapidly digested in the intestine, forming mixed micelles (colloidal dispersion), resulting in improved absorption [4]. In addition, the in vivo fate of SNEDDS depends on log P (drugs) and the solubility of drugs in SNEDDS excipients (log D of SNEDDS) because it directly impacts drug absorption [5]. Higher log P values keep the drug inside oily droplets, and more excellent solubility in SNEDDS excipients is necessary for consistent absorption and in vivo performance by avoiding drug precipitation. Drug release from liquid SNEDDS is based on a simple diffusion process and the degree of dilution in the lumen. Higher dilution volumes cause rapid drug release, resulting in erratic absorption, inconsistent serum concentrations, and unpredictable therapeutic responses, especially in FSM, which faces both solubility and permeation issues [6]. Delivering FSM using liquid SNEDDS is not ideal because of the quick emulsification in gastric fluids, improving solubility and permeation, and causing an abrupt therapeutic response. Liquid self-nano emulsifying drug delivery systems (SNEDDS) present challenges in providing controlled drug release and absorption due to their rapid dissolution in contrast to solid SNEDDS, which might provide an opportunity to address challenges [7]. Solid SNEDDS, as an evolution from liquid SNEDDS, possesses the advantages of both solid dosage forms and liquid SNEDDS. Therefore, it provides an opportunity for more controlled and sustained drug release profiles of furosemide for predictable therapeutic responses.

It was, therefore, aimed at preparing solid SNEDDS of furosemide in order to moderate the release of emulsion in GIT. For this purpose, liquid SNEDDS were prepared, subsequently adsorbed on microcrystalline cellulose, and sieved to form granules. The prepared SNEDDS formulations were further studied for stability, toxicity, lipase degradation, mucus diffusion, drug release, and ex vivo intestinal permeation in comparison to their liquid counterparts. Furthermore, diuretic activity was performed on mice to observe the difference in therapeutic response between liquid and solid SNEDDS in terms of urine volume.

## 2. Results and Discussion

### 2.1. FSM Solubility Studies

**Figure 1:**
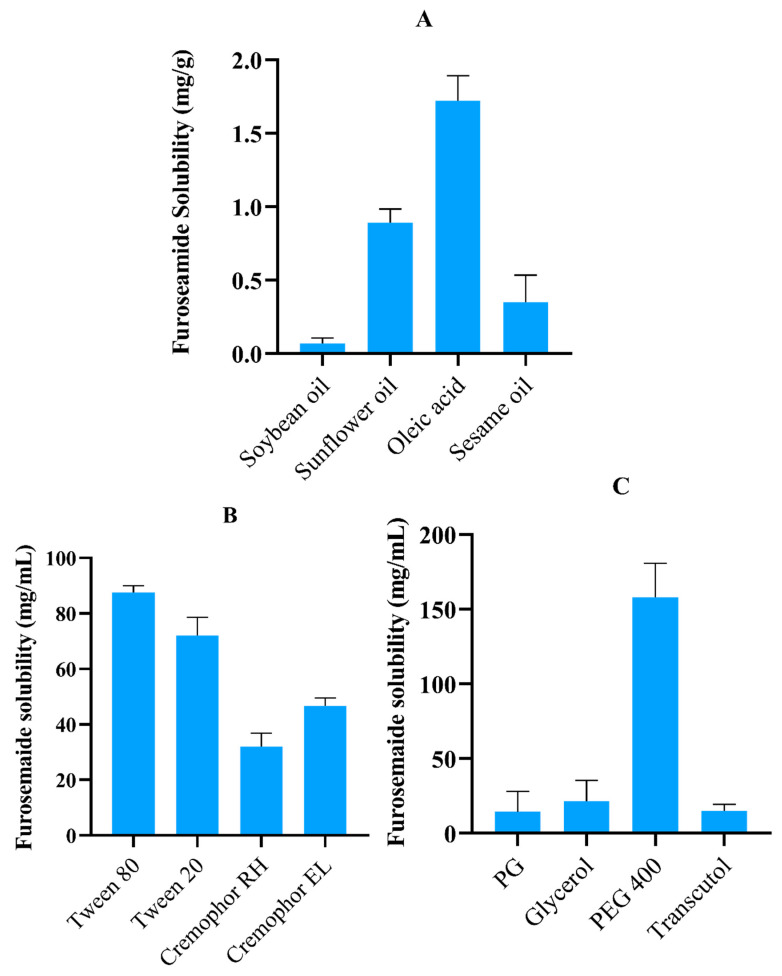
Graph displaying the solubility of FSM in (**A**) oils, (**B**) surfactants, and (**C**) co-surfactants. Values are tabulated as the mean ± SD of three experiments.

Solubility studies were performed to screen out the most suitable oil, surfactants, and co-surfactants with the highest FSM solubility. Identification of components having maximum drug solubility is of great significance as it directly correlates with drug loading efficiency [8]. Moreover, selecting appropriate excipients is also necessary for better emulsification properties. The amount of FSM soluble in various oils, surfactants, and co-surfactants is depicted in Figure 1. Among various oils, oleic acid showed a maximum solubility of 1.10 ± 0.17 mg/mL. The possible reason for maximum solubility can be referred to as the ionic interaction between the carboxylic group of oleic acid and the amino group of FSM. Results of surfactant screening revealed maximum solubilization in Tween 80 (86.22 ± 2.54 mg/mL), Tween 20 (72.30 ± 6.61 mg/mL), and Cremophor EL (47.62 ± 2.7 mg/mL). In the case of co-surfactants, PEG-400 showed a maximum solubility of 158.8 ± 9.1 mg/mL compared to other available options. Considering solubility as a critical component, oleic acid, Tween 80, Tween 20, Cremophor EL and PEG 400 were employed to develop SNEDDS for the loading and delivery of FSM [9].

### 2.2. Surfactant Emulsification Study

Surfactants having the highest solubility among their counterparts were selected and screened further for their emulsification to confirm their compatibility with oleic acid and PEG 400 (co-surfactant). Cremophor EL, Tween 80, and Tween 20 showed a percent transmittance of 99.47%, 99.12%, and 98.58% depicting good emulsifying properties. Similarly, selected surfactants demonstrated good compatibility with PEG 400, showing a percent transmittance of 99.00% (Cremophor EL), 99.20% (Tween 80), and 98.90% (Tween 20), as can be seen in Supplementary Table S1. Spontaneous emulsification of selected surfactants with oleic acid and PEG 400 is possible because of a suitable hydrophilic-lipophilic balance (HLB) > 10. Moreover, Tween 80 and Tween 20 are oleic acid-derived surfactants that provide augmented stability to the SNEDDS in cases of changing ionic strength and pH [10]. Similarly, drug-loaded surfactants also depicted that the presence of the drug did not affect the emulsifying properties of surfactants.

### 2.3. Pseudo-Ternary Phase Diagram

**Figure 2:**
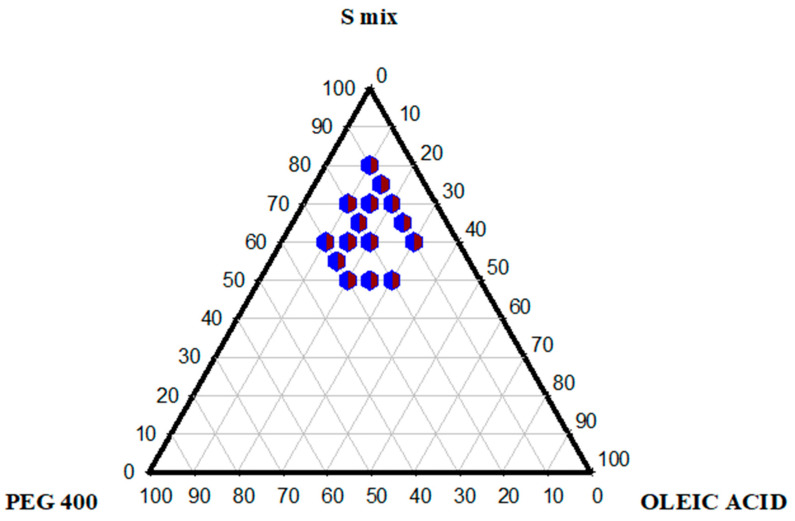
Pseudo-ternary phase diagram of oleic acid (oil), Smix (Cremophor El, Tween 80, and Tween 20), and PEG 400 (co-surfactant) depicting a nano-emulsion region (blue and red dots).

Pseudo-ternary phase diagrams were created to identify isotropic transparent self-nano emulsifying zones and get the optimized concentration of excipients for preparing SNEDDS. As shown in Figure 2, itoleic acid, Smix, and PEG 400 were used to construct the ternary phase diagram. The concentration of each surfactant in the Smix was determined by titrating varying mixtures of Smix with deionized water, and the most easily dispersible, providing clear dispersion, was selected for further studies. For constructing the ternary phase diagram, oleic acid, Smix, and PEG 400 were diluted with deionized water up to 10 mL in each sample ratio. The results provided a broader nanoemulsion region, which can be seen as a shaded region in the figure. The precise system was obtained with up to 30% oil concentration; an additional oil concentration provided turbid titration due to increased droplet size [11]. Increasing Smix concentration above 40% reduces the turbidity, and eventually, the system becomes clear above 55% Smix concentration. An increased surfactant provided a more transparent system by augmenting stabilization by lowering the oil content at the interface. Surfactants at concentrations lower than 40% destabilize the system, resulting in larger globule sizes and phase separation. Cremophor EL, Tween 80, and Tween 20 have a high HLB > 15, contributing to stable SNEDDS formulations with a transparent appearance [12]. The high HLB value surfactant Tween 80 and the low HLB value co-surfactant PEG 400 provided stability to the system in the nanoregion. These findings are from the study reported earlier by Elnaggar et al. [13].

### 2.4. Preparation of SNEDDS

A total of 58 mg of FSM was loaded per mL of SNEDDS. The increased concentration of drugs in SNEDDS is a crucial factor for in vivo performance. Moreover, drug release from SNEDDS is based on log D values, demonstrating how much the drug diffuses spontaneously from SNEDDS droplets upon dilution. Drug-loaded SNEDDS (L-SEDDS-FSM and S-SEDDS-FSM) remained transparent after incubation at 4 °C for 24 h, depicting the stability of SNEDDS preconcentrate. Similarly, no drug precipitation was observed upon dilution after incubation, which was evident from the transparent nature and absence of any turbidity [14].

### 2.5. SNEDDS Characterization

#### 2.5.1. Droplet Size, PDI, and Zeta Potential

The droplet size of SNEDDS after dilution has a decisive role in mucus diffusion and intestinal permeation. The smaller droplet size of SEEDS facilitates oily droplets reaching the epithelium swiftly through densely packed mucus gel, facilitating enhanced absorption and bioavailability. After dilution, drug-loaded L-SEDDS-FSM showed a mean droplet size of 115 ± 4.5 nm and S-SEDDS-FSM depicted 116 ± 2.6 nm. The increase in droplet size of drug-loaded SNEDDS can be attributed to the amount of drug loaded. The polydispersity index (PDI) represents size distribution; both drug-loaded and blank SNEDDS showed nearly similar PDIs of 0.12 and 0.9, respectively. Narrow PDI indicates that surfactants are more densely packed to form stable junctions at the oil-water interphase, stabilizing the oily droplets [15].

Zeta potential is another criterion for SNEDDS efficiency in vivo, attributing it to stable dispersion and interaction with glycoproteins in the mucus gel layer. L-SEDDS-FSM and S-SEDDS-FSM have zeta potentials of −5.4 ± 0.55 and −6.22 ± 1.2, respectively. The negative surface charge of both formulations is attributed to the hydroxyl group present in the employed surfactants [16]. Higher negative values of SNEDDS droplets ensure resistance to aggregation and facilitate the journey through negatively charged glycoproteins in the mucus gel layer, providing deeper penetration.

#### 2.5.2. Thermodynamic Stability

SNEDDS are in situ emulsifying systems and must be evaluated for creaming, cracking, and precipitation of dissolved drugs. Nonetheless, most SNEDDS formulations become unstable on storage for extended periods due to phase separation or drug precipitation. So therefore, prepared SNEDDS formulations were subjected to thermodynamic studies for testing against precedent instabilities. L-SEDDS-BLK and L-SEDDS-FSM were tested for stability regarding temperature and centrifugal force. No phase separation or drug precipitation were observed, as can be seen in Supplementary Figure S1 in the tested SNEDDS formulation, showing phenomenal stability after countering temperature variation and centrifugal constraint [17].

#### 2.5.3. Percentage Transmittance

Diluted SNEDDS were visually observed for any cloudiness, phase separation, and drug precipitation. L-SEDDS-FSM and S-SEDDS-FSM did not indicate phase separation or drug precipitation after dilution in a 1:100 ratio. SNEDDS were further subjected to percent transmittance, as it is critical for testing their isotropic nature and the emulsion’s nanodroplet size as well. Higher transmittance values of SNEDDS indicate optically cleared nanoemulsions having an isotropic nature. Results of both SNEDDS formulations revealed more than 98% transmittance, indicating optically cleared emulsions having nano-sized droplets, as can be seen in Supplementary Table S1. Additionally, nano-oily droplets of SNEDDS translate to higher mucus penetration, which in turn enhances uptake by the intestinal epithelium, resulting in higher bioavailability. Percent transmittance studies concluded the compatibility of selected excipients with each other, nanodroplet size, and isotropic nature of the above-tested SNEDDS formulations [18].

#### 2.5.4. pH and Dilution Stability

Oral formulations face an intense pH-changing environment, from acidic to basic, as they pass through the gastrointestinal tract. Therefore, assessing the stability of developed SNEDDS formulations at different pH values is crucial as it affects drug solubility. L-SEDDS-FSM and S-SEDDS-FSM on dilution in biological buffers with pH 1.2 and 7.4 showed no signs of drug precipitation. Moreover, in these buffer systems, SNEDDS were further tested for their robustness in dilutions in an increased fashion, i.e., 100 and 1000 times. Both SNEDDS formulations emulsified spontaneously without any sign of phase separation or drug precipitation, indicating robustness to dilution, as can be seen in Supplementary Figure S2. Drug release from SNEDDS is based on diffusion; upon dilution, unstable formulations change appearance from transparent to translucent, representing phase separation or cloudiness indicating drug precipitation [19]. Conclusively, the stability of developed SNEDDS against such high dilutions represents the optimum concentration of oil, surfactants, and co-surfactants in a formulation necessary to stabilize oily emulsion droplets in a GIT environment [20].

#### 2.5.5. ATR-FTIR

The ATR-FTIR spectra of furosemide showed us N-H stretching at 3399 cm, N-H stretching associated with the sulfonyl group at 3349–3284 cm, the carbonyl group at 1671 cm, S = 0 stretching between 1141–1322 cm, and N-H bending at 1563 cm. FTIR of L-SEDDS-FSM and S-SEDDS-FSM showed peaks in the same region, showing FSM in the formulations as can be seen in Supplementary Figure S3.

### 2.6. Hemolysis Studies

**Figure 3:**
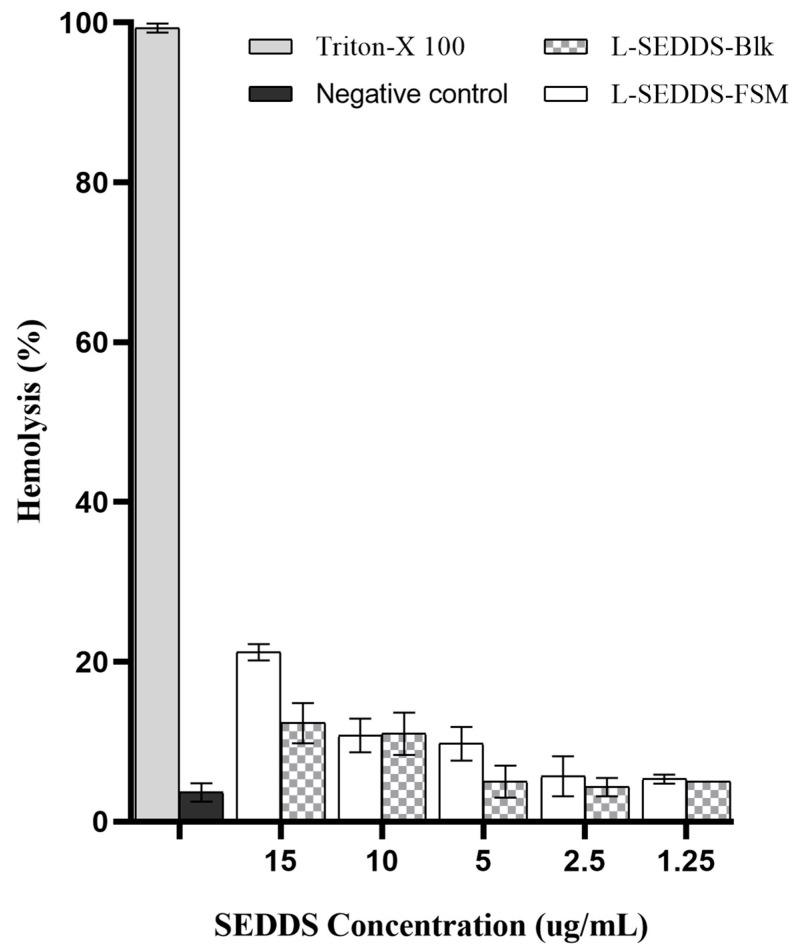
Hemocompatibility assay of L-SEDDS-Blk and L-SEDDS-FSM in concentrations of 1.25 µL/mL to 15 µL/mL using Triton X-100 as a positive control and PBS buffer pH 7.4 as a negative control. All values are taken in triplicate as a mean ± SD.

In vitro, a hemolysis assay is a reliable technique to assess biocompatibility by evaluating the interaction between SNEDDS and lipid cell membranes. Erythrocytes accurately depict the lipid bilayer membrane surrounding endosomes for assessing cellular toxicity [21]. Surfactants in SNEDDS compromise cell membrane integrity because of their strong solubilization capacity to dissolve lipid bilayer-enclosing cells. As shown in Figure 3, SNEDDS has depicted no hemolytic activity at a concentration of 1.25 µL/mL while, upon increasing concentration, showing maximum hemolysis of 16% at 15 µL/mL. Moreover, at higher concentrations, drug-loaded L-SEDDS-FSM showed minor hemolysis differences compared to SNEDDS without the drug, suggesting that the incorporation of FSM is relatively safe. All formulations showed less than 16% hemolysis, falling in the relatively safe zone. The dilution factor of SNEDDS used in this assay is somewhat higher than the actual in vivo environment, omitting toxicity concerns. The biocompatible blend of non-ionic, less-irritant excipients in SNEDDS also accounts for decreased hemolysis [22]. Moreover, excipients used in the formulation of SNEDDS have a well-established safety profile approved by the FDA.

### 2.7. In Vitro Lipase Assay

**Figure 4:**
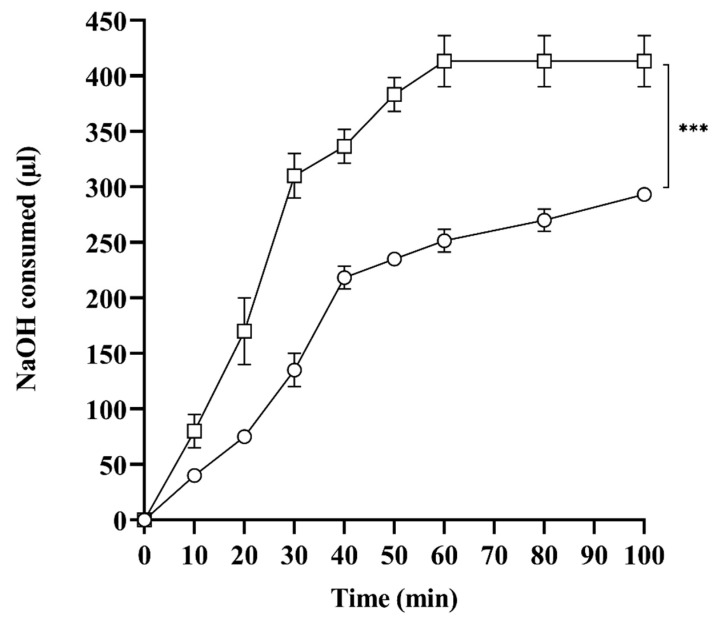
Total NaOH consumed by L-SEDDS-FSM (□) and S-SEDDS-FSM (○) upon dilution to digestion medium containing lipase over a time period of 100 min. All values are taken in triplicate as a mean ± SD.

SNEDDS are exposed to lipase in the lumen, digesting lipids into uni-and multi-vesicles and mixed micelles, affecting drug solubilization and absorption. In vitro, lipolysis studies provide significant information for defining SNEDDS stability by mimicking the most crucial phases in the absorption of lipid-based formulations, such as dispersal and digestion [23]. Lipase hydrolyzes triglycerides to one or two monoglycerides and releases fatty acids, measured by titrating with NaOH to predict the rate of lipolysis. Therefore, the amount of NaOH consumed is directly related to the degree of SNEDDS hydrolysis, which releases fatty acids. As shown in Figure 4, L-SEDDS-FSM, under the influence of lipase, consumed 400 µL of NaOH compared to S-SEDDS-FSM, which consumed 220 µL of NaOH, depicting 1.8-fold more extended stability than liquid SNEDDS. Spontaneous emulsification of liquid SNEDDS provides the immediate opportunity for lipase to hydrolyze and release fatty acids, resulting in quick digestion. On the contrary, the preconcentrate lipid formulation embedded deep inside a solid SNEDDS matrix requires an additional release step for emulsification. Moreover, liberation from the polymeric matrix is always subject to slow diffusion, providing a slow emulsification pattern. In addition, SNEDDS preconcentrate within the polymeric matrix is not readily accessible to lipase, providing the best explanation for decreased degradation and enhanced stability. Gradual unwrapping of the preconcentrate from the polymeric matrix slows down drug diffusion, providing an additional advantage in preventing erratic absorption of FSM and avoiding unpredictable therapeutic responses. The slow clearance of lipid formulations from the polymeric matrix offers an additional benefit in drug delivery. Hence, recently developed solid SNEDDS are less accessible to lipase for cleaving ester linkages, shielding lipid formulations against in vivo digestion and erupted drug release.

### 2.8. In Vitro Drug Release Studies

**Figure 5:**
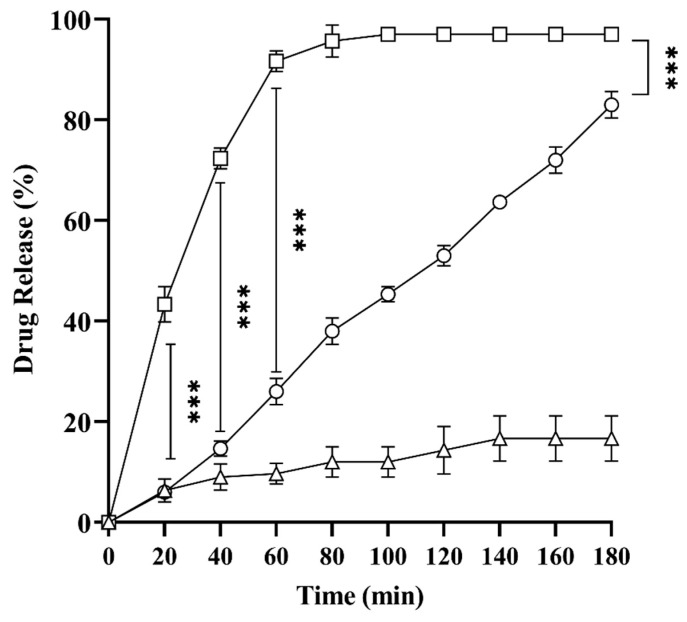
Drug release profiles of L-SEDDS-FSM (□), S-SEDDS-FSM (○), and pure drug FSM (∆) as a control for a period of 180 min. All values are taken in triplicate as a mean ± SD.

In vitro drug release studies were performed to assess the pattern of drug escape from solid SNEDDS in comparison to liquid SNEDDS. Previously, studies established that drug release from liquid SNEDDS follows simple diffusion, depending on factors like the log P of the drug, the amount of the drug in SNEDDS, and the degree of dilution [24]. As shown in Figure 5, upon dilution, L-SEDDS-FSM showed 44% drug release in 10 min, while drug release from S-SEDDS-FSM was only 5%. Similarly, FSM only showed 3–7% dissolution in the first 60 min after dispersion. L-SEDDS-FSM releases over 90% of the drug in the first 30 min. In contrast, S-SEDDS-FSM depicted 63% of drugs in 180 min, presenting a sustained pattern of drug release. Sudden drug release from liquid SNEDDS is problematic and responsible for the unpredictable therapeutic response, especially for drugs like FSM. Moreover, several studies have shown poor in vitro-in vivo correlation with such erratic drug release in liquid SNEDDS [23]. The possible reason for S-SEDDS-FSM’s delayed release is the slow eruption of preconcentrate from the microcrystalline matrix and gradual emulsification [25]. The effectiveness of SNEDDS in dissolution is thought to be dependent on two factors: first, the capacity of the self-emulsifying formulation to create an emulsion with consistent fine particle size droplets, and second, the capacity of SNEDDS to deliver the drug in a solubilized and highly distributed form, thereby avoiding the dissolution step. Solid SNEDDS achieved both goals, making the release curve more linear and sustained.

### 2.9. Mucus Diffusion Studies

**Figure 6:**
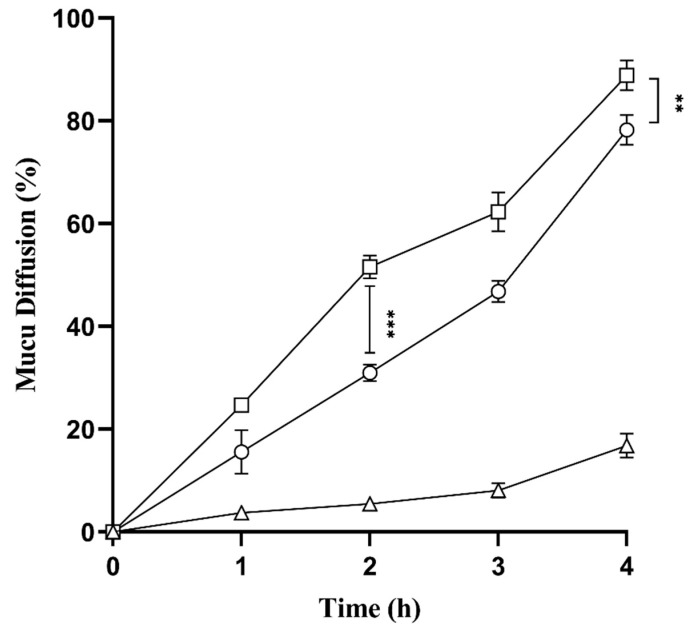
Mucus diffusion studies of L-SEDDS-FSM (□), S-SEDDS-FSM (○), and FSM (∆) as a control over a time period of 4 h. Experiments were performed in triplicate as a mean ± SD.

The interaction between mucus and newly developed SNEDDS (L-SEDDS-FSM and S-SEDDS-FSM) was evaluated using goat intestinal mucus. Figure 6 shows that bsoth SNEDDS formulations exhibited 5.2 times higher mucus diffusion than FSM. The higher diffusivity of SNEDDS is due to using pegylated surfactants, Cremophor EL and PEG 400, as co-surfactants [26]. These surfactants and co-surfactants form an interface between oily droplets of SNEDDS and a highly charged mucus gel layer, facilitating the diffusion of encapsulated drugs. Mucus is a dynamic barrier that limits the passage of particles not only based on their size but also through ionic interaction. The Zeta potential of the SNEDDS droplets has a significant impact on mucus diffusion properties as well. Developed SNEDDS formulations have negative zeta potential, creating a repulsive force to push deeper into the SNEDDS while interacting with mucus glycoproteins [27]. Moreover, it can be concluded from the results that even after SNEDDS solidifies, mucus permeation efficiency remains nearly the same as that of liquid SNEDDS.

### 2.10. Ex Vivo Intestinal Permeation Studies

**Figure 7:**
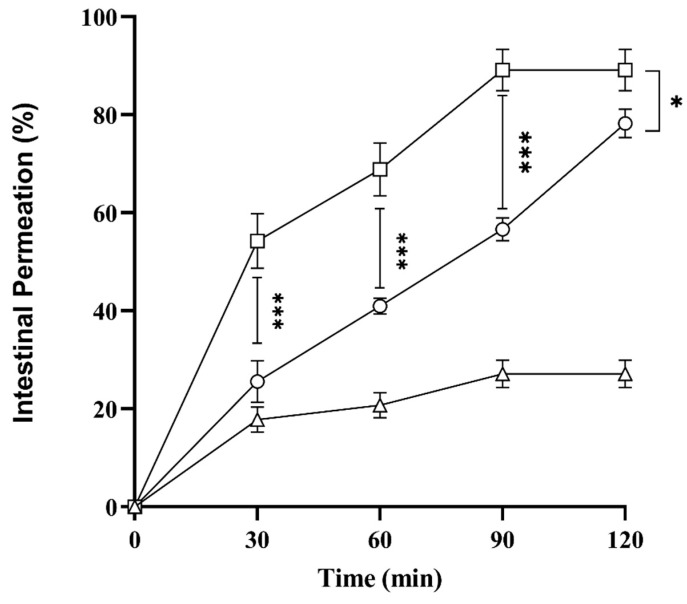
Ex vivo permeation studies were performed using freshly excised goat small intestine on L-SEDDS-FSM (□), S-SEDDS-FSM (○), and FSM (∆) as a control for a period of 2 h. All values are taken in triplicate as a mean ± SD.

Oral bioavailability of drugs relies on solubility and permeation through the intestine, which is also an essential factor. Therefore, to assess intestinal drug permeation via SNEDDS, ex vivo permeation studies were carried out using goats’ intestines. Results in Figure 7 showed that L-SEDDS-FSM and S-SEDDS-FSM exhibited 89 ± 4.2% and 78.27 ± 2.92% drug permeation in 120 min compared to FSM, which is only 27.12 ± 2.81%. This shows that SNEDDS increases the solubility and enhances the intestinal permeation of encapsulated drugs. Additionally, increased permeation is also in line with the mucus diffusion efficiency of SEEDS compared to drugs without SNEDDS. Surfactants and co-surfactants in SNEDDS irreversibly disrupt the intestinal epithelium, thus increasing intestinal permeation and bioavailability.

Moreover, SNEDDS also inhibit the Pg-P efflux mechanism due to glycol moieties such as cremophor EL and Tween 80, increasing the influx of drugs encapsulated in oily droplets [28]. Paralleled, S-SEDDS-FSM showed a sustained curve of intestinal permeation owing to the slow release of SNEDDS pre-concentrate from the microcrystalline cellulose matrix. L-SEDDS-FSM revealed rapid irregular permeation of FSM, which is mainly responsible for erratic absorption and unpredictable therapeutic responses.

### 2.11. In Vivo Studies in Mice

Furosemide is a potent loop diuretic, and diuresis is either negative or has no correlation with plasma drug concentration; therefore, the amount of urine output is an efficient method to quantify therapeutic response [29]. Because of this, in vivo studies were performed by measuring therapeutic response in terms of urine output. The diuretic studies were conducted for 5 h in mice. As shown in Figure 7, L-SEDDS-FSM has depicted a quick upsurge in urine output of 1550 ± 56 µL in the 4th h of the study compared to S-SEDDS-FSM, providing urine output of 969 ± 29 µL. At the 5th hour of the study, L-SEDDS-FSM showed a sudden drop in urine volume to 1355 ± 78 µL, providing an unreliable therapeutic response, while S-SEDDS-FSM gradually increased the urine volume up to 1360 ± 42 µL, depicting a predictable therapeutic response. In comparison, FSM dispersed in water showed minor diuretic activity and thus produced the least diuretic volume of 752 ± 2.8 µL. Diuretic activity findings dictated that S-SEDDS-FSM provided prolonged and delayed diuretic effects of FSM.

**Figure 8:**
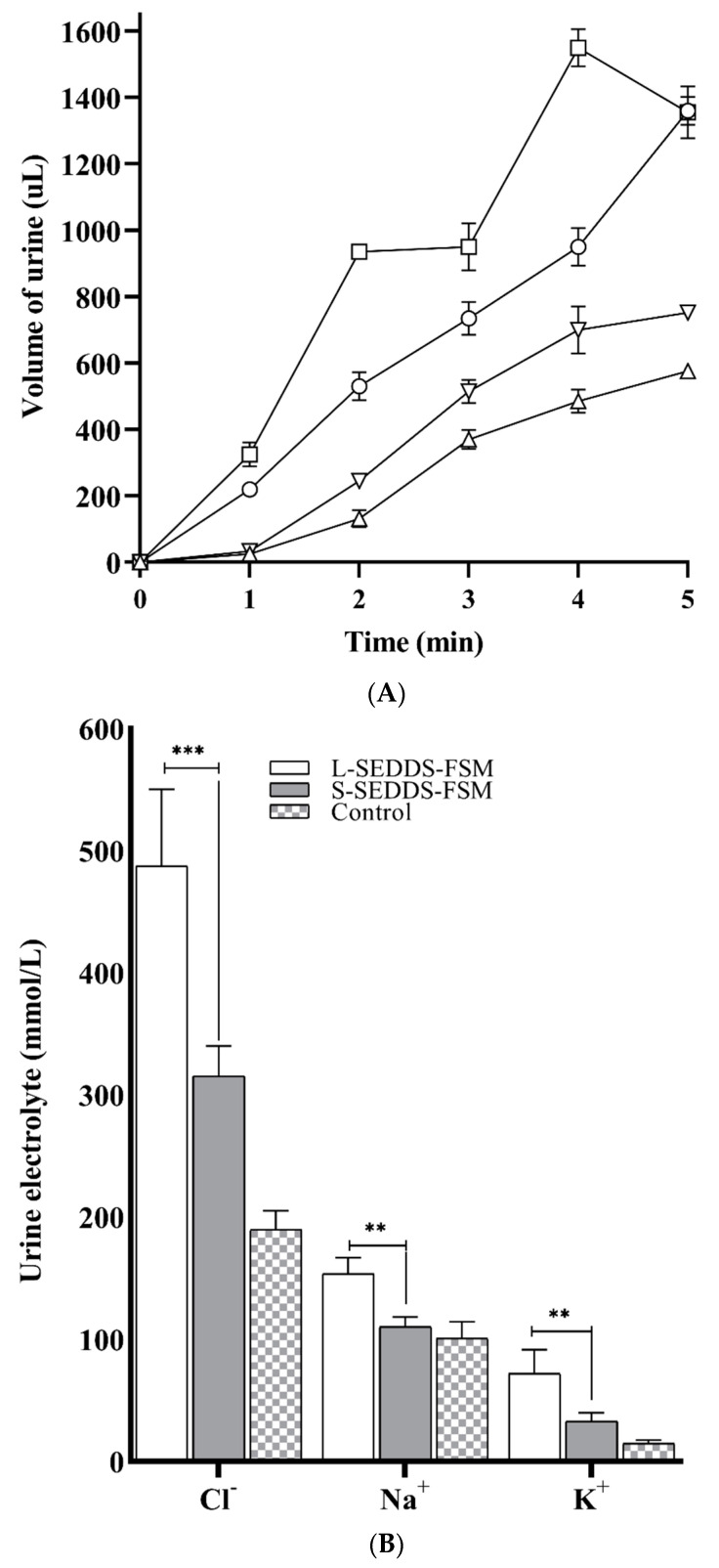
(**A**) Volume of urine measured after administration of L-SEDDS-FSM (□), S-SEDDS-FSM (○), and FSM (∇) to mice using normal saline (∆) as control over a time period of 5 h. Experiments were performed in triplicate and calculated as mean ± SD. (**B**) Accumulative electrolyte concentration measured after administration of L-SEDDS-FSM, S-SEDDS-FSM, and FSM to mice using buffer as control over a period of 5 h. All values were calculated as the mean ± SD after three experiments.

Erratic absorption of furosemide can lead to unpredictable serum blood concentrations, likely affecting electrolyte balance [30]. Figure 8A represents electrolyte excretion, i.e., Na^+^, K^+^, and 2Cl^−^, after administration of both SNEDDS formulations containing FSM, normal saline, and FSM. L-SEDDS-FSM showed the highest excretion of electrolytes in comparison to Sol-SEDDS-FSM and FSM. It was observable that L-SEDDS-FSM excreted 2Cl^−^ 520 ± 34, Na^+^ 144 ± 12, and K^+^ 69 ± 13 at the 5th h of the study. In comparison, S-SEDDS-FSM released 2Cl^−^ 430 ± 14, Na^+^ 111 ± 15, and K^+^ 58 ± 15 in urine, respectively. The volume of urine excreted by L-SEDDS-FSM and S-SEDDS-FSM was nearly the same at 5 h, but there is a difference in electrolyte excretion, as clearly illustrated in Figure 8A. The results summarize that S-SEDDS-FSM caused less depletion of electrolytes in comparison to L-SEDDS-FSM. This response might be due to thecontrolled absorption of FSM from S-SEDDS-FSM, owing to the sustained release of preconcentrate and slow emulsification in the gut. Because of erratic absorption, furosemide, a high ceiling loop diuretic known for the excretion of electrolytes, especially 2Cl^−^, Na^+^, and K^+^, causes electrolyte imbalance, causing hypochloremia, hyponatremia, and hypokalemia, respectively. Such an extreme imbalance of the electrolytes has significant clinical complications in patients on such diuretics.

Na+ is a critical external variable for maintaining blood pressure and fluid retention. The arterial blood pressure is positively impacted by increased Na+ excretion [31]. In this study, L-SEDDS-FSM demonstrated considerable natriuretic effects as compared to S-SEDDS-FSM, potentially bringing blood pressure to a significantly low level and causing hypotension. Conclusively, a BCS IV diuretic furosemide must be consistent in solubility and intestinal permeation for a predictable diuretic response to avoid hypovolemic shock and electrolyte imbalance. The results established that FSM via S-SEDDS-FSM was sustained in absorption and produced predictable diuresis, causing less depletion of electrolytes compared to its counterpart for a specific therapeutic response, as can be seen in Figure 8B.

## 3. Material and Methods

### 3.1. Materials

Furosemide, lipase, minimum essential medium, oleic acid, Tween 80^®^, Tween 20^®^, polyethylene glycol, and glycerol were purchased from Sigma Aldrich (St. Louis, MO, USA). Gattefossé, Neuilly sur-Seine, France, gifted Cremophor EL^®^, Transcutol HP^®^, and Cremophor RH 40^®^. All other reagents used were of analytical grade.

### 3.2. Methods

#### 3.2.1. Solubility Studies

Solubility studies of FSM in various oils (soybean oil, sunflower oil, castor oil, oleic acid, and sesame oil), surfactant (Cremophor EL, Cremophor RH 40, Tween 80, Tween 20), and co-surfactants (Propylene glycol, Glycerol, PEG 400, and Transcutol HP) were assessed. µ Afterwards, vials were centrifuged at 2000 rcf for 10 min, and the supernatant was collected. 100 µL of supernatant was dissolved in 1900 µL of 0.1 M NaOH, and the amount of dissolved FSM was measured using a UV-spectrophotometer at 274 nm [32].

#### 3.2.2. Investigation of Surfactant Emulsification

Surfactants selected based on having the highest solubility of FSM were further investigated for their emulsification efficiency. For this purpose, the oil phase and surfactant were mixed in a ratio of 1:1, followed by heating at 35–40 °C to obtain a homogenized mixture. 200 µL of the resulting mixture was diluted in 10 mL of deionized water to form an emulsion, which was observed by analyzing the number of inversions of the volumetric flask. After an incubation period of 1 h, percent transmittance was measured using a UV-visible spectrophotometer at 638 nm, taking deionized water as a blank [33].

#### 3.2.3. Ternary Phase Diagram

To optimize the concentration of oil, surfactant, and co-surfactant to form SNEDDS pre-concentrates that, upon dilution, form nanoemulsions. For this purpose, excipients (oils, surfactants, and co-surfactants) with the highest FSM solubility were selected. Oleic acid was chosen as oil, Tween 80, Tween 20, Cremophor EL as surfactants, and PEG 400 as co-surfactant. First, the quantity of surfactants was calculated by titrating 10–90% concentration mixtures of cremophor EL, Tween 80, and Tween 20 using 10 mL deionized water to determine the proportion for Smix. A total of 100 µL of these surfactant mixtures were diluted using 10 mL of deionized water and visually inspected for turbidity or translucency. Smix ratios showing no turbidity were selected for further studies. Selected Smix ratios, oleic acid, and PEG 400 were combined in different concentrations to make 100%. Each pre-concentrate mixture with a variable component concentration was prepared using a thermomixer at 300 rpm at 40 °C for 15 min. 100 µL of the resulting SNEDDS formulation were diluted with 10 mL of distilled water and observed visually for the appearance of a milky or transparent mixture, indicating the formation of either micro-emulsion or nano-emulsion, respectively. The observations were then tabulated, and percentages of oil, surfactant, and co-surfactant were calculated and plotted using Sigma plot software 15.0 to indicate our region of interest, a nano-emulsion region [34].

#### 3.2.4. Preparation of SNEDDS Formulations

##### Preparation of Liquids SNEDDS

Excipients with the highest FSM solubility were selected and mixed in pre-determined ratios, as described in Table 1. For this purpose, L-SEDDS-Blk was developed by combining 200 µL of oleic acid with S_mix_ comprising 400 µL, 200 µL, 100 µL, and 100 µL of chremophor El, Tween 80, Tween 20, and PEG 400, respectively, at 50–60 °C and vortexed (1000 rcf) for 4–5 min to ensure proper mixing of all excipients, which is then used as blank in further experiments [35]. Furthermore, L-SEDDS-FSM was formulated using the same proportions as L-SEDDS-Blk, except containing 53 mg of FSM [36].

##### Preparation of Solid SNEDDS

For the preparation of solid SNEDDS (S-SEDDS-FSM), a preconcentrate of liquid SNEDDS (L-SEDDS-FSM) was used as a liquid phase, while microcrystalline cellulose was employed as a solid carrier. Briefly, 1 mL of L-SEDDS-FSM preconcentrate was added dropwise to 1 g of microcrystalline cellulose and thoroughly mixed with the help of a pestle and mortar until a wet mass was produced. The resulting mixture was passed through sieve no. 10, and the obtained granules were dried at 40 ℃ and stored in a desiccator for further studies [37].

#### 3.2.5. SNEDDS Characterization

##### Droplet Size, Polydispersity Index, Zeta Potential

Globule size, polydispersity index, and zeta potential of L-SEDDS-Blk. L-SEDDS-FSM and S-SEDDS-FSM were performed on a zeta analyzer (Malvern, Malvern, UK). For this purpose, 100 µL of F_Blank_ and F_FSM_ were diluted in 1 mL of deionized water using a vortex mixer to form nanoemulsions. At the same time, 200 µg of S-SEDDS-FSM was vortexed with 1 mL of deionized water and centrifuged at 3000 rcf for 5 min. The supernatant was collected for measurement. All measurements were taken at 25 ℃ in triplicate [38].

##### Thermodynamic Stability

SNEDDS are thermodynamic dispersion; we expect them to remain stable after exposure to variable temperatures. Therefore, prepared SNEDDS preconcentrates (liquid and solid) were assessed for their thermodynamic stability by incubating them at 4 °C and 40 °C (heating-cooling cycles), followed by incubation at −20 °C and 25 °C (freeze–thaw cycles) for at least 48 h. Afterwards, SNEDDS were reconstituted in a 1:100 ratio using deionized water and centrifuged at 9660 rcf for 15 min. The resulting samples were observed for any drug precipitation and phase separation [39].

##### Percent Transmittance

F_FSM_ was reconstituted at 1:100 using deionized water, while 200 µg of S-SEDDS-FSM were diluted in 1 mL of deionized water and centrifuged to collect the supernatant. Dilutions were observed visually for any turbidity. For further confirmation, dilutions were measured at 638 nm using UV–a visible spectrophotometer to measure percent transmittance, employing distilled water as a blank.

##### Stability and Robustness to Dilution

The stability and efficiency of prepared SNEDDS were evaluated at pH 1.2 and 7.4. To perform studies, 100 µL of L-SEDDS-FSM and 200 µg of S-SEDDS-FSM were diluted in 10 mL of HCl buffer (pH 1.2) and phosphate buffer pH (7.4) to form nanoemulsion. Visual observation was recorded for the transparency of the emulsion [40].

##### FT-IR Analysis

To confirm the encapsulation of FSM into SNEDDS, FT-IR analysis of pure FSM, L-SEDDS-FSM, and S-SEDDS-FSM was performed by ATIR-FTIR (Alpha 1-Bruker, Karlsruhe, Germany). Measurements were taken by recording absorbance in the 4000–500 cm^−1^ range.

#### 3.2.6. Hemolysis Assay

To evaluate the toxicity of the prepared SNEDDS, a human red blood cell (RBC) concentrate donated by Nishtar Hospital Blood Bank was used to perform the hemolytic assay. RBC concentrate was obtained by centrifuging blood at 1677 rcf for 10 min and removing supernatant plasma. Afterwards, the RBC concentrate was diluted by suspending 0.556 mL of RBC concentrate in 0.994 mL of sterile Dulbeccos PBS (pH 7.4), prepared freshly before each test. To obtain an equivalent amount of whole blood (EWB), RBC suspension was 50 times diluted with sterile Dulbeccos PBS (pH 7.4). Diluted blood appeared turbid on visual inspection, and RBCs settled down when left undisturbed, indicating no cell lysis. Different concentrations of L-SEDDS-Blk and L-SEDDS-FSM ranging from 1.25–15 µL/mL were prepared using 10 µL of each test sample in 190 µL of diluted blood. 10 µL of 0.1% triton X served as a positive control, 10 µL of Dulbeccos PBS in 190 µL of diluted blood was taken as a negative control, and 100 µL of SNEDDS dispersed in 900 µL of PBS was taken as a blank. All test samples, including positive and negative controls, were incubated at 37 °C for 1 h under gentle stirring. After incubation, samples were centrifuged at 1677 rcf for 2 min, and 1 mL of aliquots were taken to perform UV at wavelength 420 nm. The extent of the hemolysis percentage was then calculated by using the formula [21].

$$CapPercentage\;hemolysis=\frac{absorbance\;\left(test\right)-absorbance\;\left(negative\right)}{absorbance\;\left(positive\right)-absorbance\;\left(negative\right)}\times 100$$

#### 3.2.7. In Vitro Lipolysis Studies

Dynamic in vitro lipolysis of the SNEDDS was performed to assess their fate in physiological conditions. The digestion medium comprises 35.5 mL of digestion buffer, 50 mM tris-HCl, 150 mM NaCl, and 5 mM CaCl_2_, but does not contain bile salts due to their inhibitory effect on pancreatic lipase.It was continuously stirred at 1000 rpm and maintained at 37 °C. The medium was set up the day before the lipolysis assay and left to equilibrate at 37 °C overnight via adjusting pH and volume. Pancreatic lipase suspension was prepared by dispersing 1 g of lipase in 5 mL Tris buffer. Subsequently, the enzyme suspension was centrifuged at 2415 rcf for 7 min, the supernatant was collected, and the pH was adjusted to 6.5 via 0.5 M NaOH. Freshly prepared lipase suspension was kept in an ice bath at a temperature between 6 and 8 °C to avoid degradation. Test samples were prepared by adding 100 µL of L-SEDDS-FSM and 200 µg of S-SEDDS-FSM in 9 mL of digestion buffer. The pH meter was calibrated using standard buffers of pH values that were almost neutral. Lipolysis was started by adding 1 mL of lipase suspension to L-SEDDS-FSM and S-SEDDS-FSM. After adding 1 mL of lipase, suspension samples were incubated for 1 h. A decrease in pH was noticed due to the liberation of FFA, which indicates the digestion of SNEDDS. This decrease in pH was readjusted by using 0.5 M NaOH. The extent of SNEDDS digestion was measured by calculating the total volume of NaOH consumed [41].

#### 3.2.8. Mucus Diffusion Studies

The intestines of a freshly slaughtered goat were collected and incised longitudinally. Waste debris and other food materials were gently removed by washing with cold water. The mucus was gently collected from the intestinal surface using a scrapper to avoid the pealing of epithelial tissues. For purification, 50 g of mucus was dispersed, 250 mL of 0.1 M sodium chloride was used, and it was stirred for 1 h at 4 °C, followed by centrifugation at 6000 rcf for 2 h in an ice bath. After centrifugation, the supernatant was discarded, and clear mucus was again suspended in an ice bath in 250 mL of 0.1 M sodium chloride for 2 h. Purified mucus obtained after centrifugation was stored at 8 °C for further use.

2 mg of FSM dispersed in 0.1 M NaOH was taken as a control compared to L-SEDDS-FSM and S-SEDDS-FSM. For performing mucus diffusion, 100 µL of L-SEDDS-FSM and 200 µg of S-SEDDS-FSM were dispersed in 1 mL of 0.1 M PBS having pH 6.8. In the Franz diffusion cell, the donor chamber was covered with 1 g of mucus, and the acceptor chamber was filled with 3 mL of 0.1 M PBS (pH 6.8). The permeation process was started by introducing SNEDDS dilution in the donor chamber. At an interval of 60 min, 500 µL of aliquot was taken from the acceptor chamber of each sample and replaced by an equal amount of fresh PBS pH 6.8 at 37 °C. Observations were made by calculating the amount of drug in each aliquot using a UV-spectrophotometer at a wavelength of 274 nm. The percentage of drug diffused through the mucus was calculated as the ratio of drug present in the acceptor compartment to the 100% reference value after each sample removal [42].

#### 3.2.9. In Vitro Drug Release

The in vitro release of L-SEDDS-FSM was performed using phosphate buffer pH 6.8 as the dissolution medium. 200 µg of S-SEDDS-FSM and 100 µL of L-SEDDS-FSM were dispersed separately into 100 mL of dissolution medium, stirring (100 rpm) at 37 °C for 2 h. 1 mL of aliquot from each sample was withdrawn at 5, 10, 30, 50, 60, and 120 min and replaced by an equal volume of dissolution medium. A double-beam UV spectrophotometer analyzed samples at a wavelength of 274 nm. A blank correction containing a 1:1000 dilution of L-SEDDS-Blk was used to avoid assay interference with the formulation’s component. The percentage of FSM released from SNEDDS was calculated and plotted as a function of time.

#### 3.2.10. Ex Vivo Permeation

Ex vivo studies were conducted on young goats’ duodenal portion of the small intestine obtained from the local slaughterhouse. Freshly excised duodenum was washed with phosphate buffer pH, 6.8. A 2.54 cm^2^ fragment was cut and placed between the acceptor and donor chambers of Franz diffusion cells in such a manner that the bottom side interfered with the reception media and the apical portion facing donor compartment. 2 mg of FSM dispersed in 0.1 M NaOH solution was taken as a control. 100 µL of L-SEDDs-FSM and 200 µg of S-SEDDS-FSM were dispersed in 1 mL of 0.1 M PBS with pH 6.8. The acceptor chamber was filled with 3 mL of PBS pH 6.8, and the donor chamber was filled with each SNEDDS dilution. Approximately 500 µL of aliquots from each sample were withdrawn from the acceptor chamber at intervals of 0, 1, 2, 3, and 4 h and replaced by an equal amount of fresh PBS (pH 6.8) at 37 °C. The amount of drug permeated was determined by using a UV-spectrophotometer at a wavelength of 274 nm. The percentage of drug permeated was calculated as the ratio of drug permeated in the acceptor compartment in sample treatment to the 100% reference value after each sample removal.

#### 3.2.11. In Vivo Studies Experimental Design in Mice

Adult male albino rats weighing 25–35 g were selected for the study. Standard laboratory conditions, i.e., 25 ± 1 °C temperature, 55 ± 5% humidity, 12/12 h light and dark cycle, were maintained along with free access to tap water and standard laboratory mice food. Animals were kept in metabolic cages for 24 h before the start of experimentation. Each animal was kept in a separate cage for adaptation, had free access to water, and fasted for 8 h before starting an experiment. All mice were given normal saline (0.9% NaCl) at 0.75 mL per 10 g of mouse weight to ensure every individual’s uniform salt and water load. Each mouse was then subjected to the treatment as follows: Groups 1 and 2 were given L-SEDDS-FSM and S-SEDDS-FSM, respectively, at 10 mg/kg. Group 3 was given FSM dispersed in water at the same dose, whereas group 4 was kept as control and was given a vehicle for reconstitution (water) at a dose of 2 mL/100 g of body weight. Urine was collected at 1, 2, 3, 4, and 5 h intervals, taking into account both volume and frequency. Electrolytes, including sodium, potassium, and chloride, were also analyzed [43].

#### 3.2.12. Statistical Analysis

All experiments were conducted three times and mean ± SD is calculated. Moreover, Student T test was applied and significance *p* ≤ 0.05 is calculated. The steric stars in the figures represent the level of significance, i.e., (*) Significant, (**) very significant and (***) highly significant.

## 4. Conclusions

Within this study, liquid and solid SNEDDS of furosemide were prepared and characterized for their toxicity studies, lipase stability, drug release, mucus penetration, and in vivo diuretic activity. The study has demonstrated that S-SEDDS-FSM showed 1.8-fold more stability against lipase degradation and provided 1.6-fold more sustained drug release compared to liquid SNEDDS. Moreover, S-SEDDS-FSM showed 5-fold increased mucus penetration and 2.8-fold higher intestinal permeation compared to FSM. S-SEDDS-FSM gradually increased diuresis in mice, while L-SEDDS-FSM resulted in an irregular diuresis pattern because of their spontaneous drug release. Additionally, S-SEDDS-FSM, due to the slower release of FSM, provided consistent oral absorption in mice, resulting in controlled diuresis and a predictable therapeutic response. According to these results, the solidification of SNEDDS proved to be a promising strategy to overcome spontaneous drug release and the unpredictable therapeutic response associated with liquid SNEDDS formulation, improving furosemide’s efficacy and safety profile.

## Figures and Tables

**Table 1:** Composition of different SNEDDS formulations having oil, surfactant, and co-surfactant concentrations, along with size and charge distribution.

Chemicals	FSM (mg)	Avicel	Oleic Acid (µL)	Cremophor EL (µL)	Tween 80 (µL)	Tween 20 (µL)	PEG 400 (µL)	Zeta Size (nm)	Zeta Potential (mV)
L-SEDDS-Blk		-	200	400	200	100	100	116 ± 4	−6.3
L-SEDDS-FSM	53	-	200	400	200	100	100	115 ± 2.6	−5.4
S-SEDDS-FSM	53	1:1	200	400	200	100	100	116 ± 4.3	−4.3

## Data Availability

Data is contained within the article and Supplementary Materials.

## Supplementary Materials

The following supporting information can be downloaded at: https://www.mdpi.com/article/10.3390/ph17040500/s1, Figure S1: Thermodynamic stability assessment of L-SEDDS-BLK and L-SEDDS-FSM after a freeze-thaw cycle to assess the stability of the formulation; Figure S2: Robustness to dilution and stability studies after diluting L-SEDDS-FSM and S-SEDDS-FSM in 1:100 and 1;1000 indicating no precipitation or phase separation; Figure S3: ATR-FTIR spectrum of FSM, L-SEDDS-FSM and S-SEDDS-FSM showing incorporation of FSM inside both formulations; Table S1: Percent transmittance values of a combination of Surfactant + oils, Surfactant + co-surfactant, L-SEDDS-FSM and S-SEDDS-FSM after emulsification.
