# Supplementary material for: Solid Self-Nanoemulsifying Drug Delivery Systems of Furosemide: In Vivo Proof of Concept for Enhanced Predictable Therapeutic Response
Source: Pharmaceuticals (Basel). 2024 Apr 14;17(4):500. doi: 10.3390/ph17040500 (PMC11053802; doi:10.3390/ph17040500)
Supplement: Supplementary file 1 [file pharmaceuticals-17-00500-s001.zip › pharmaceuticals-2947179-supplementary.pdf]

Supplementary Data

Supplementary Table S1 Percent transmittance values of a combination of Surfactant + oils, Surfactant + co-surfactant, L-SEDDS-FSM and S-SEDDS-FSM after emulsification.

Formulations	Figures	UV results
Tween 20+ oil	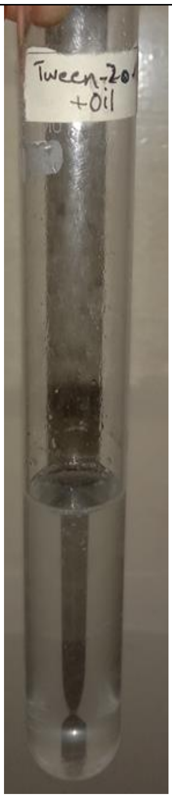	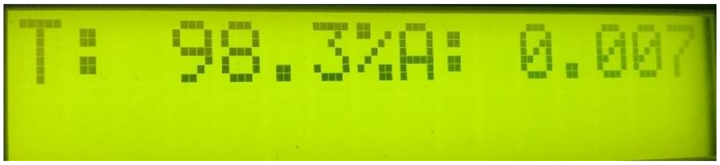

Tween 80+ oil	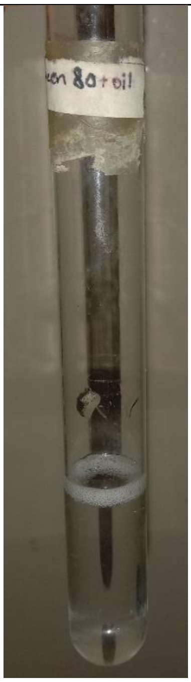	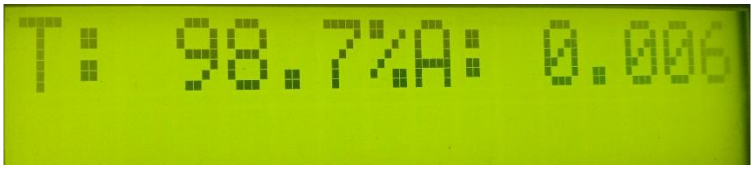
Cremophor EL+ oil	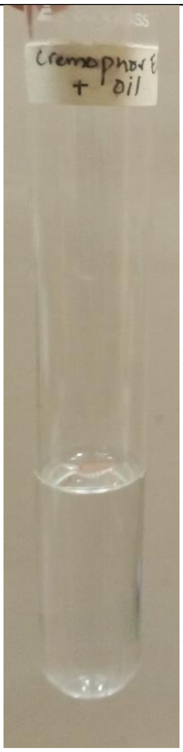	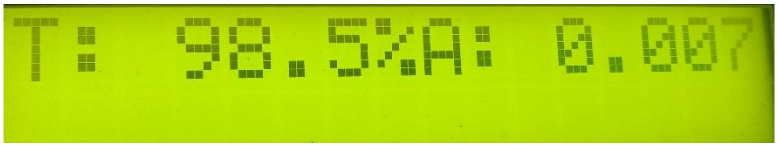

Tween 20+ PEG	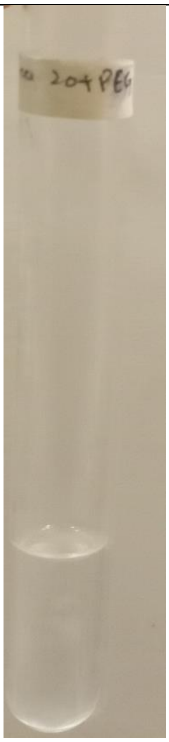	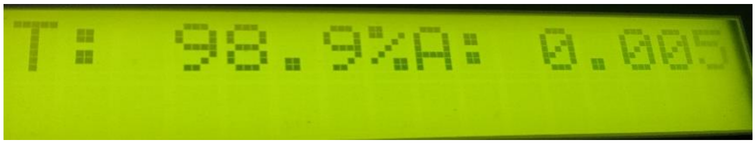
Tween 80+ PEG	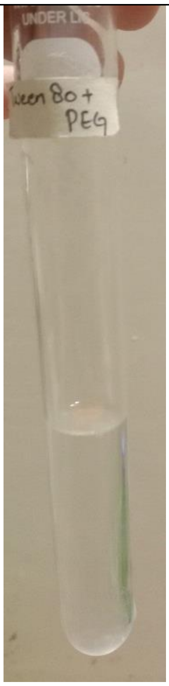	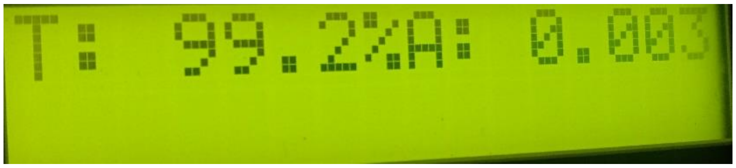

Cremophor EL + PEG	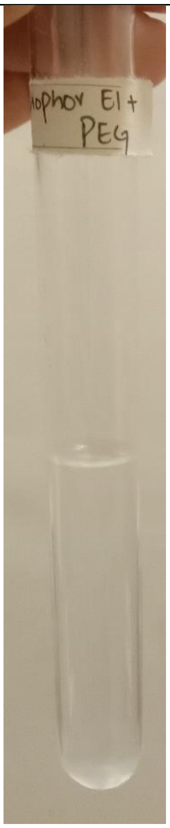	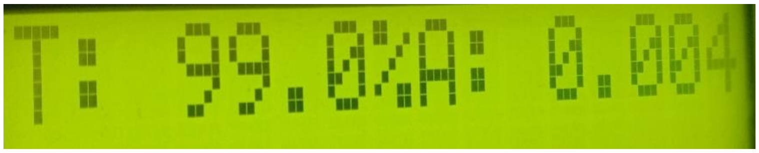
L-SEDDS-FSM	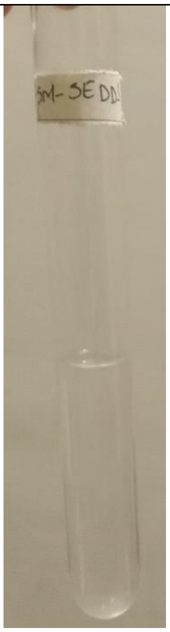	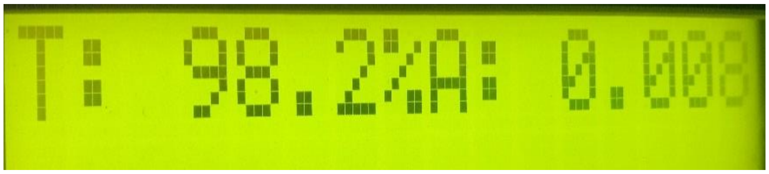

S-SEDDS-FSM	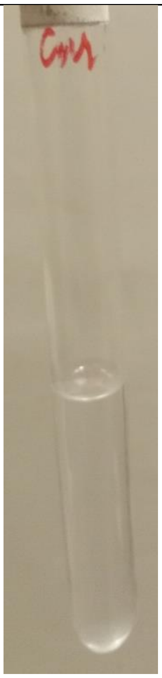	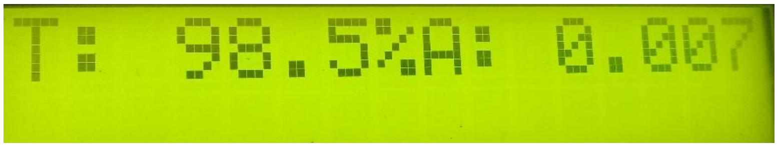

Supplementary Figure S1 Thermodynamic stability assessment of L-SEDDS-BLK and L-SEDDS-FSM after a freeze-thaw cycle to assess the stability of the formulation.

At -20˚C	At 25˚C
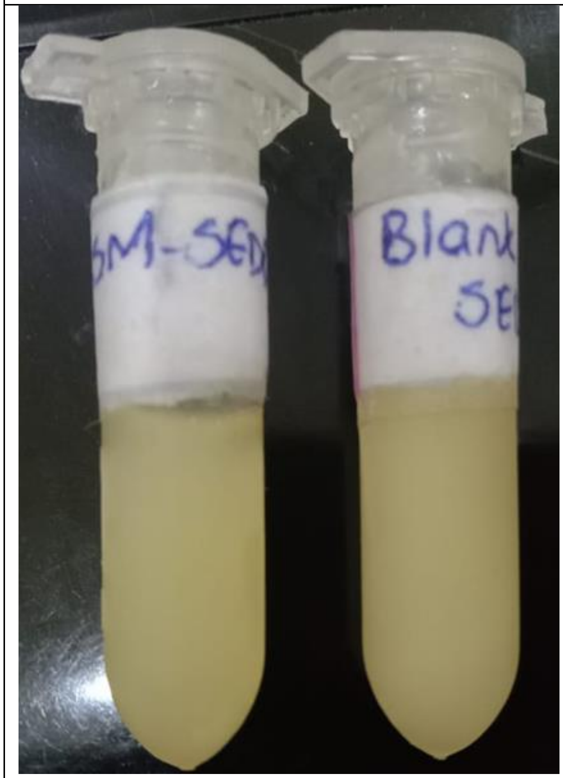	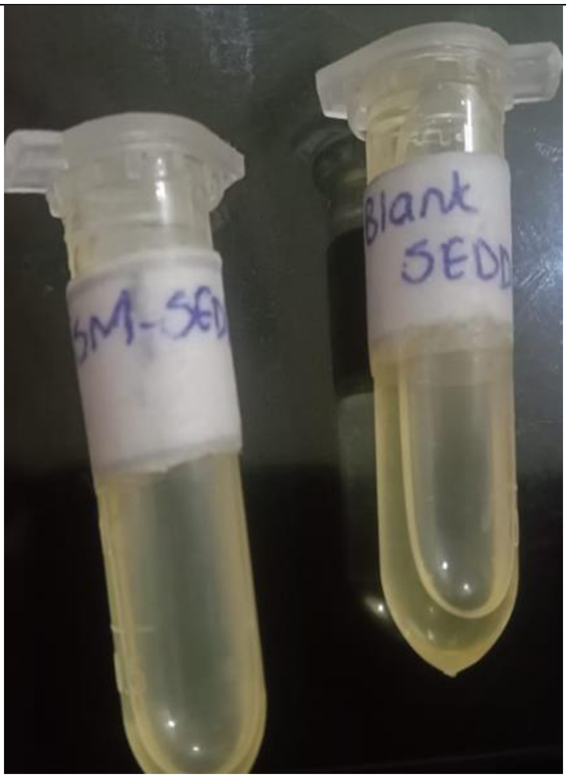

Heading cooling cycle:

At 4˚C		At 40˚C
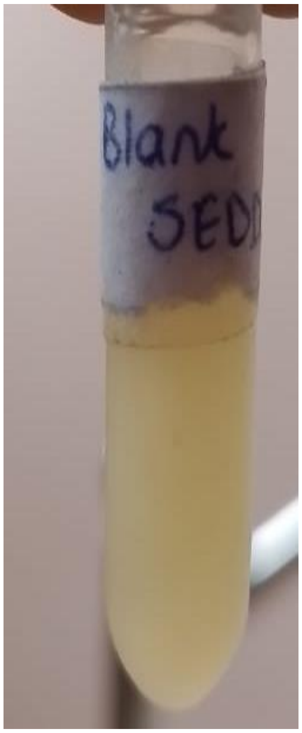	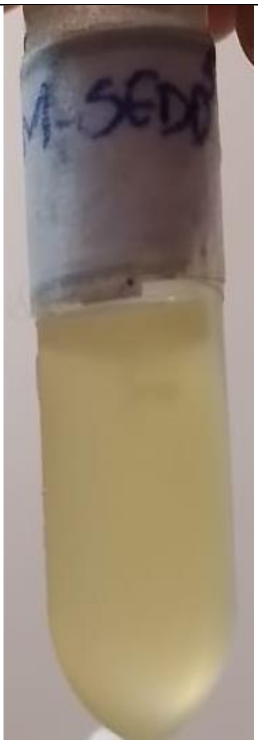	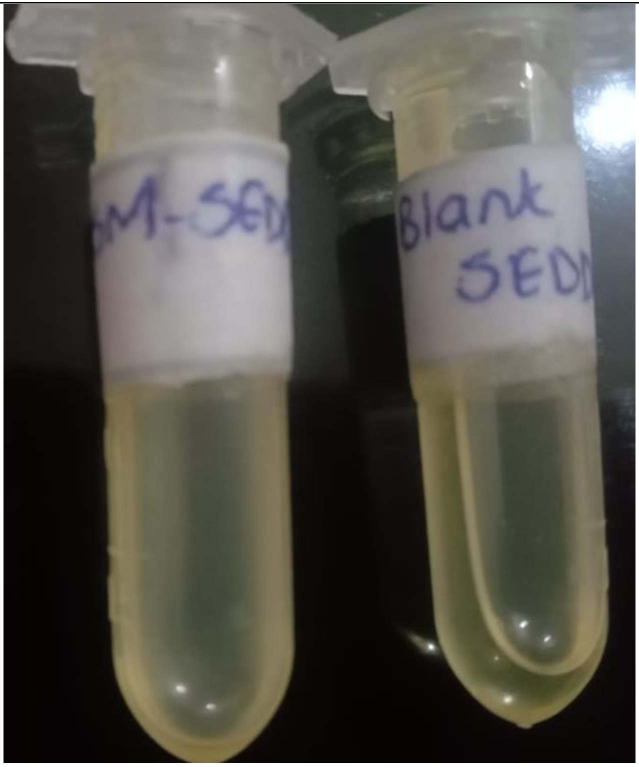

Supplementary Figure S2 Robustness to dilution and stability studies after diluting L-SEDDS-FSM and S-SEDDS-FSM in 1:100 and 1;1000 indicating no precipitation or phase separation.

**Dil. 1:100**	**Dil. 1:1000**
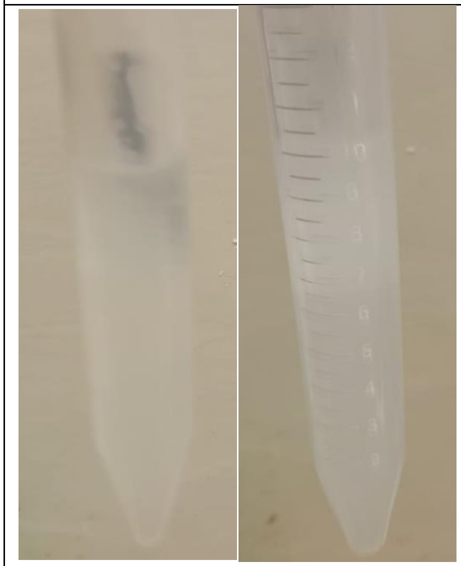<br>*L-SEDDS-FSM* *S-SEDDS-FSM*	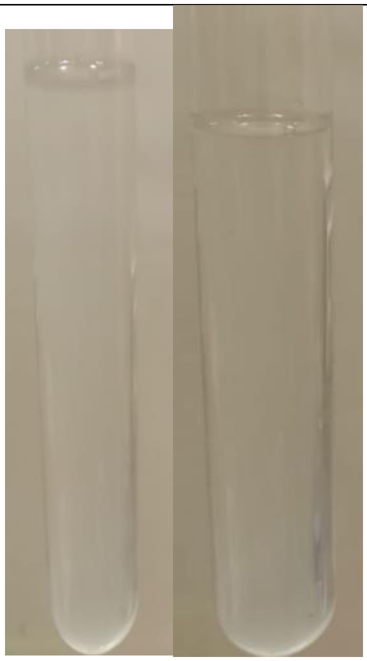<br>*L-SEDDS-FSM* *S-SEDDS-FSM*

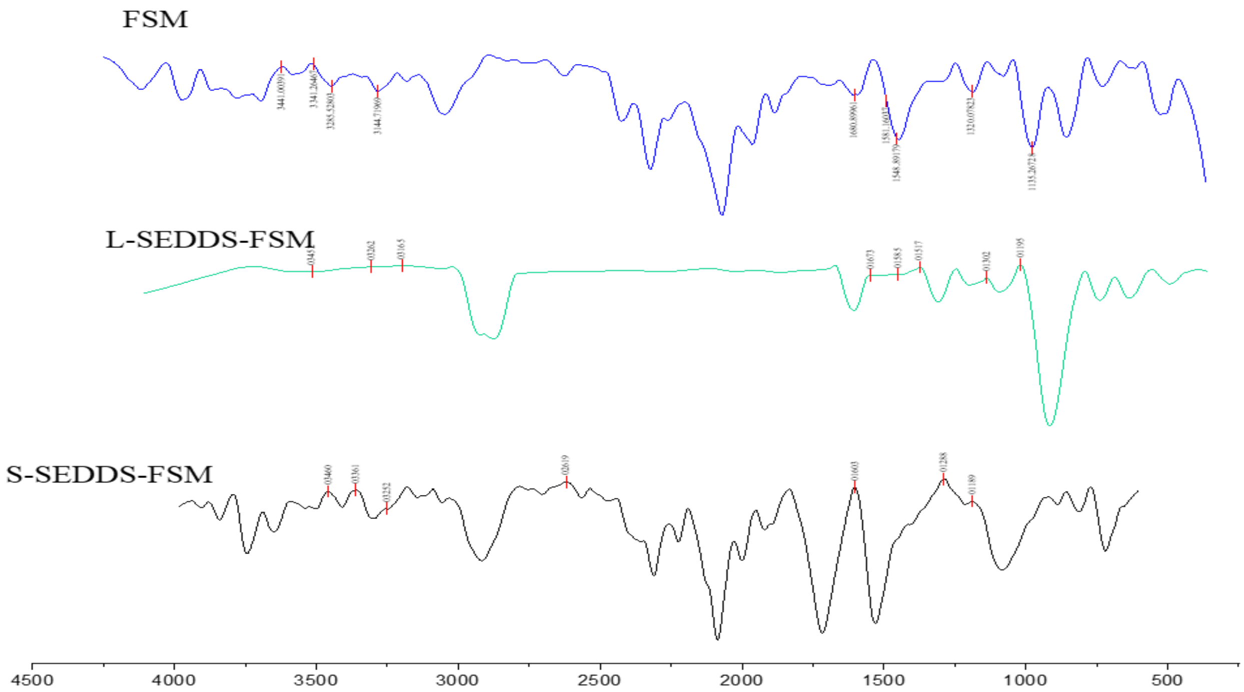

Supplementary Figure S3. ATR-FTIR spectrum of FSM, L-SEDDS-FSM and S-SEDDS-FSM showing incorporation of FSM inside both formulations.
